# Dissemination and implementation of an educational tool for veterans on complementary and alternative medicine: a case study

**DOI:** 10.1186/s12906-016-1297-4

**Published:** 2016-09-02

**Authors:** Rachel Forster Held, Susan Santos, Michelle Marki, Drew Helmer

**Affiliations:** 1 VA New Jersey Healthcare System, East Orange, NJ USA; 2 Department of Medicine, Rutgers University-New Jersey Medical School, Newark, NJ USA

## Abstract

**Background:**

We developed and disseminated an educational DVD to introduce U.S. Veterans to independently-practiced complementary and alternative medicine (CAM) techniques and encourage CAM experimentation. The project’s goal was to determine optimal dissemination methods to facilitate implementation within the Veteran’s Health Administration.

**Methods:**

In the first phase, the DVD was disseminated using four methods: passive, provider-mediated, active, and peer-mediated. In the second, implementation phase, “champion” providers who supported CAM integrated dissemination into clinical practice. Qualitative data came from Veteran focus groups and semi-structured provider interviews. Data from both phases was triangulated to identify common themes.

**Results:**

Effective dissemination requires engaging patients. Providers who most successfully integrated the DVD into practice already had CAM knowledge, and worked in settings where CAM was accepted clinical practice, or with leadership or infrastructure that supported a culture of CAM use. Institutional buy-in allowed for provider networking and effective implementation of the tool. Providers were given autonomy to determine the most appropriate dissemination strategies, which increased enthusiasm and use.

**Conclusions:**

Many of the lessons learned from this project can be applied to dissemination of any new educational tool within a healthcare setting. Results reiterate the importance of utilizing best practices for introducing educational tools within the healthcare context and the need for thoughtful, multi-faceted dissemination strategies.

## Background

Educational tools for patients are constantly created, disseminated and implemented across healthcare settings, with the ultimate aim of improving health outcomes. Dissemination involves planned efforts to spread experimentation with or adoption of the tool, while implementation refers to efforts to integrate it into usual care. Healthcare education tools come in many formats and can be disseminated using different strategies. According to systematic reviews, evidence comparing the effectiveness of dissemination strategies is mostly equivocal [1–3]. However, passive dissemination, such as handing out or mailing printed materials, is less effective than active dissemination strategies and does not show an effect on patient outcomes [2].

Multi-component dissemination strategies show more powerful effects than any single strategy alone [2–4]. There is some evidence that characteristics of certain dissemination strategies may enhance ease of use, such as the inclusion of how-to materials, tailored toolkits, or skill training for providers involved in dissemination [4].

Greenhalgh et al. [5] proposed three broad system antecedents for innovation, i.e. characteristics of a facility that facilitate the successful dissemination of educational tools. These system antecedents include (1) institutions that are large and mature enough to incorporate changes, since smaller, newer institutions focus on more basic growth and have fewer resources; (2) preexisting knowledge base within the institution, to better integrate new materials; and (3) a receptive context for change, including appropriate leadership within an organization. They also identified factors that enhance implementation once a system is ready for dissemination, including assigning decision-making to frontline medical teams and having good internal communication, thereby allowing providers to communicate dissemination strategies with one another.

This paper details the dissemination and implementation of an educational tool called the STAR Well-Kit (SWK), a DVD designed to introduce U.S. Veterans to Complementary and Alternative Medicine (CAM) practices. The VA Office of Patient Centered Care & Cultural Transformation funded the development of the SWK to promote CAM modalities as a more patient-centered approach reflecting Veteran interest in these services [6]. Given the geographic dispersion of VA healthcare facilities and the limited on-site expertise available at many VA healthcare facilities at the time, the SWK focused on modalities which can be practiced independently, such as yoga, meditation, and breathing. The goals of the SWK were to raise awareness by educating Veterans about the benefits of CAM practices using Veteran testimonials and to allow them to try several brief demonstration practices. The DVD consisted of 85 min of content divided into four major segments and included closed captioning. The major segments included an introduction to CAM for Veterans, interviews with Veterans on their CAM experience, brief CAM practices to try along with the DVD (specifically, soft belly breathing, guided meditation, qigong, chair yoga, and hatha yoga), and views from the provider community regarding the benefits of CAM for Veterans. With increased awareness, Veterans could then seek additional CAM opportunities, whether through multimedia recordings or classes within their VA or community.

In line with the best-practices above, SWK dissemination involved multi-component strategies, and was distributed with “tip sheets” for both Veterans and their providers. Once the SWK and its accompanying materials were designed, the question remained as to optimal implementation techniques given the institutional structure of the VA, the needs of particular Veterans and providers, and the fact that CAM is a relatively novel and nontraditional approach to improving wellness. To determine best practices for dissemination and implementation of the SWK, we considered many factors, including the available resources of the VA, the availability and involvement (or lack thereof) of providers, and the variability of VA facilities’ existing CAM-related infrastructure.

This paper describes the dissemination and implementation of the SWK, including successful strategies and lessons learned. The value of understanding SWK dissemination and implementation process is not exclusive to this project. Rather, it highlights important lessons and strategies that may be applied to the broad dissemination and implementation of many healthcare-related educational tools.

## Methods

The SWK was disseminated and implemented over two phases. The first, dissemination phase, focused on spreading experimentation with the SWK and assessing the best settings and methods for adoption. The second, implementation phase was an effort to integrate the SWK into usual care on a broader scale. Methods for the two phases are described separately.

### Dissemination phase

During the initial phase, 725 Veterans were given SWK packets containing an instruction letter, pre- and post-viewing surveys with return envelopes, and a focus group invitation. All dissemination took place within the catchment area of the Veterans Affairs-New Jersey Health Care System (VA-NJHCS), which covers northern and central New Jersey. The ambulatory service settings were chosen with a goal of ensuring participant diversity in terms of age, deployment experience, and mental and physical health. Beyond being a Veteran, there were no inclusion or exclusion criteria for participants receiving the SWK, as participation was anonymous. However, providers, peers and team members who distributed the SWK were encouraged to discuss the need for access to a DVD player or computer with DVD drive.

Four dissemination methods were used: (1) Active Dissemination: A research team member explained the study in waiting rooms in one of three clinic settings: primary care, mental health, and clinics for recently separated Iraq or Afghanistan Veterans (“post-deployment clinics”). The researcher distributed packets and explained the contents to interested Veterans. (2) Passive Dissemination: Along with an explanatory poster, unattended copies of the SWK packet (usually ten at a time) were left in waiting rooms in the three selected clinic types for Veterans to take at their own behest. Interested Veterans were expected to follow the instructions in the cover letter. A member of the research team monitored packets to see how many had been taken. (3) Healthcare Provider-Mediated Dissemination: We asked healthcare providers of many types from the three selected clinic types to provide the packet to patients in the context of their routine clinical visit. Providers who volunteered were advised to use their discretion to decide how and to whom to present it. (4) Peer-mediated Dissemination: A community-based peer support and outreach group staffed by Veterans offered the packet to Veteran clients they interacted with by phone, in person, or at group outreach events. Funding was provided for this group to be involved in the dissemination. Peer-counselors were given talking points for disseminating packets.

Five Veteran focus groups were conducted by research team members, with 43 Veterans participating. Participants had all received a SWK packet and called or returned a mailing stating their interest in joining a focus group. The purpose of the focus groups was to assess Veteran awareness, knowledge and perceptions of CAM, to pilot test the SWK content, and obtain feedback on the various SWK dissemination techniques used, as well as other possible strategies. Some limited information on the packaging of the DVDs was also addressed. Finally, Veterans were asked to discuss possible next steps of using the DVD and any barriers. Two trained moderators led the groups and each session was recorded. At the end of each group, the moderator and at least one observer/research assistant discussed top line observations and noted minor additions to the discussion guide for subsequent groups. Waivers of informed consent were approved by the VA-NJHCS IRB given the anonymous and low-risk nature of the research. The focus group audio recordings were transcribed and de-identified by assigning a numeric ID to each participant in the focus groups. Using the grounded theory approach, “discovery of theory from data--systematically obtained and analyzed,” [7] the research team developed a code book and 2–3 researchers independently coded each group using the software “NVivo 8.” Differences in coding were reconciled to attain consensus.

The coded text was then reviewed in an iterative fashion from which themes emerged. Once themes were developed the researchers went back through the coded text to find quotes that supported or best illustrated the theme.

During the initial phase, providers who agreed to disseminate SWK packets were asked to return a questionnaire with their impressions of the SWK and their experiences disseminating it. They were also asked to volunteer for semi-structured interviews to further assess their approaches to dissemination. Forty-seven providers agreed to disseminate, 14 returned surveys, and eight participated in semi-structured interviews. NJHCS IRB waived the informed consent of provider interviews.

Data from this project is potentially available from the corresponding author subject to VA’s policies and regulations on privacy and information security.

### Implementation phase

This phase involved dissemination throughout the VA healthcare system and collecting program evaluation data to assess effectiveness. Data from Veteran focus groups suggested that engaging providers in SWK dissemination would be beneficial. The low initial response from volunteer providers prompted the focus on “champion” providers in the implementation phase. Champion providers were physicians, psychologists, social workers, nurses and physician assistants who had existing knowledge of CAM and its nascent role in VA healthcare. These providers were targeted as more likely to implement the SWK successfully. They were identified from across the national VA system based on involvement in VA Office of Patient Centered Care and Cultural Transformation (OPCC & CT) programs, or involvement with the War Related Illness and Injury Study Center (WRIISC).

Champions were emailed information about the SWK and asked if they were interested in disseminating DVDs. Interested providers watched DVDs and completed semi-structured interviews assessing their views of the DVD, whether they would like to disseminate copies, and if so, how and to whom. Interviews were recorded and key segments transcribed. Fifteen hundred of the DVDs (28 %) were mailed with program evaluation surveys on views of CAM for Veterans to complete, with attached return envelopes. Interested providers received DVDs to disseminate and a “dissemination suggestions tip sheet” with information on optimizing dissemination, based on feedback from the initial phase. Six weeks after DVDs were mailed, providers gave follow-up information by phone or email. Follow-up questions assessed whether dissemination was going according to original plan, whether it had changed and why, what barriers they had encountered, and what feedback or conversations they had with Veterans about the SWK. Engaged providers were offered additional DVDs to distribute. Some providers requested additional DVDs to share with their colleagues, which we provided.

### Analysis

The current paper focuses on qualitative information from providers and Veterans during both phases of the project. In addition, quantitative results were calculated from the tracking systems used by the research team and are interspersed in the results to provide additional context. During the dissemination phase, we used the grounded theory approach, involving “discovery of theory from data” [6]. We developed coding schemes to code transcripts, from which themes emerged. Two researchers independently coded transcriptions, and differences were reconciled to attain inter-coder reliability. During the implementation phase, data collected for program evaluation purposes were analyzed using the basic coding scheme from the dissemination phase. Subsequently, the research team triangulated the dissemination and implementation phase data to identify and organize themes that emerged from Veteran focus groups, provider semi-structured interviews, and other communications with providers.

## Results

### Descriptive dissemination data

During the initial dissemination phase, 725 SWK packets with enclosed DVDs were distributed. Table 1 shows the number of packets disseminated by each method, and the number and response rate of pre and post-SWK surveys returned by each method.

**Table 1:** Number of Veteran packets disseminated and returned, by dissemination method

	Number disseminated	Pre-surveys returned	Post-surveys returned	Pre to post response rate
Provider	351	14 (4 %)	6 (2 %)	43 %
Passive	79	2 (2 %)	2 (2 %)	100 %
Direct	173	73 (42 %)	29 (17 %)	40 %
Peer	120	45 (38 %)	19 (16 %)	42 %
Overall	725	134 (18 %)	56 (8 %)	42 %

**Fig. 1:**
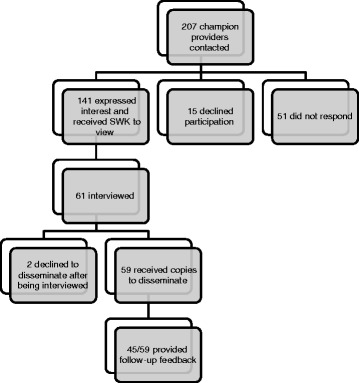
Champion provider contact and involvement during implementation phase. (SWK = Star Well Kit)

**Fig. 2:**
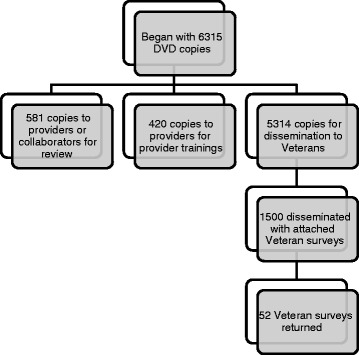
DVD dissemination during implementation phase

Figure 1 shows the number of providers involved in the second, “champion” implementation phase. Figure 2 shows the number of DVDs disseminated during this phase.

### Qualitative results

Qualitative results are presented together for both phases, since many common themes were observed.

#### Effective dissemination methods engaged patients, and when relevant, engaged their providers

##### Passive dissemination methods did not motivate veterans to try the SWK

Passive dissemination efforts were included early on, as a more traditional and low-cost dissemination approach. These efforts were stopped early due to lack of follow-up response from those who took packets from waiting rooms. Although packets in waiting rooms disappeared quickly, of the 79 packets distributed with this method, only two pre-surveys (2 %) and two post-surveys (2 %) were returned. Patients may have taken packets from waiting rooms without interest or understanding of the content. It is likely that many of them never watched the DVD.

##### Having educational materials on CAM disseminated in a one-on-one interaction, by either peers or researchers, was initially found to be most successful

Compared with more passive forms of dissemination, dissemination through direct conversations with researchers or peers led to more interest in receiving the SWK, and to a higher rate of survey return (42 % for pre-surveys, and 17 % for post-surveys), suggesting these Veterans were more likely to watch the DVD. These strategies afforded the Veterans greater opportunity to engage with the idea of CAM. Researchers or peers talked with Veterans and explained the SWK project, giving them an opportunity to decline further engagement without taking a packet, to ask questions, or to hear positive things about CAM that may convince them to view the DVD and complete the surveys. In Veteran focus groups, some stated that peer dissemination increased their comfort with the SWK presentation. *“It was [disseminated at] a large group meeting… and it's all Vietnam veterans, Korean, all veterans, and we talk about… VA healthcare.”*

##### Point-of-care dissemination by providers was unsuccessful unless the providers were in appropriate positions to integrate the SWK into care

Despite provider dissemination in a point-of-care setting, early efforts to distribute the SWK through providers were unsuccessful, with a 2–4 % patient survey response rate for those who received the SWK from their providers. Only 30 % of providers returned surveys about their experiences with dissemination, suggesting low engagement on their part. Although providers doing initial dissemination agreed to participate, they were handed packets for Veterans with little explanation or follow-up.

Many providers gave no follow-up data on their methods of dissemination during the initial phase. Due to busy schedules or lack of involvement, it is possible that many distributed the SWK without discussion. Some providers who did offer qualitative feedback during our initial dissemination (8 out of 47) described having difficulty integrating conversation about the SWK into their sessions, which may have been on unrelated topics. For example:

*A social worker working with Veterans who recently returned from Iraq and Afghanistan spent her session time focusing on “more immediate, pragmatic issues”, and stated the Veterans were “confused” if she changed the topic to the SWK at the end of the session.*

Additionally, several providers expressed concerns about limited time with Veterans. One stated, *“I think that a lot of staff will say, ‘I don't have time for this.’”*

#### Providers poised for optimal dissemination had preexisting CAM knowledge and were positioned to incorporate change

##### For successful, sustained dissemination, providers need to have an interest and knowledge of the product

While our initial dissemination focused on Veterans, we found that providing the tool to clinical champions was a better implementation strategy. Veteran focus group participants stated they would like their healthcare providers to engage with them about CAM. One stated, *“I gave [my doctor] a copy of [the SWK] so she could kind of see what I can do besides those pills that she prescribes.”* In addition, having providers disseminate the educational allows for a more sustainable effort than a time-limited effort by research staff or by Veteran peers hired for the project.

During the interviews with champions prior to dissemination, we found that these providers had a better understanding of the material, which likely helped them more effectively communicate the tool’s value to their patients. Provider champions gave positive feedback about the SWK; many liked the Veteran testimonials of their experiences, and the variety of techniques offered. They reported they could more easily integrate its use into their existing treatment approaches and were more motivated to find time for it. Champion providers also described greater confidence to respond to patient questions and engage with their patients about the content in a way that other providers might not. Finally, many providers shared that they tried to foster Veteran interest because of their own belief in the effectiveness of CAM practices.

##### Targeting providers based on their resources and their work environment and culture was more effective than targeting by their specialty or expertise

At first, we had assumed that mental health providers would be keen on using the SWK, since CAM can aid with stress and anxiety. However, mental health providers were not as involved as expected early on. During the first phase, only 12 mental health providers volunteered to disseminate the SWK packets (25 % of 41 providers), and only three (25 %) returned surveys about the SWK. While mental health providers may use CAM within their own clinical practice, some reported that they viewed some CAM modalities as established clinical practice and did not feel the need to enhance CAM interest among their patients. In the second, implementation phase, only ten percent (seven out of 66) of providers were in mental health departments, as we targeted providers with access to CAM resources and a culture of CAM promotion, rather than targeting by profession or specialty per se.

#### Fostering successful implementation strategies

##### Successful, sustained implementation of the SWK benefited from broader availability of CAM resources at the local facility. These resources increased providers’ comfort level and allowed them to provide referrals for additional services, thereby increasing provider interest in SWK dissemination and veteran uptake

While targeting champions to disseminate the SWK was an attempt to start with those already interested, provider interest was not sufficient to maximize SWK dissemination. Feedback received from Veterans in the focus groups suggested they were more apt to try a practice if they had access to services where it could be better learned and replicated; one stated, *“You’re going to do better with a group or somebody that is more advanced.”* Having existing CAM programs or educational opportunities in place within a facility, and having administrative or leadership supports for CAM were associated with greater provider success in disseminating the SWK. Many champions had some connection to CAM programs at their local VA. In several follow-up interviews, providers reported Veterans expressing interest in adopting a practice and “asking them how or where they could do so;” one “asked for additional yoga resources.” Many providers were able to follow up by referring Veterans to programs offered within their own facility or in the local community.

##### Effective dissemination techniques either allowed veterans to connect with a provider and engage with the material, or targeted veterans who were already in a setting where they may be more open to learning about CAM

Champion providers reported that many Veterans were interested in the DVD. In these cases the providers often took time in their patient encounter to discuss the DVD content and how the Veteran might use the sample practices and tools in the DVD, or even to watch a few minutes of it together. Watching the DVD together creates a joint learning opportunity and may be especially valuable for patients without access to a DVD player. Successful one-on-one dissemination sometimes took place during visits focused on overall wellness (e.g., with a psychologist in a health and wellness center), possibly because Veterans were more open to learning about CAM when they perceived it to fit into the purpose of the visit. Providers had success disseminating the DVD in one-on-one settings when they saw patients for regular visits and were therefore able to follow-up more, such as regular occupational therapy visits.

##### Having institutional buy-in and support for CAM led to increased provider interest in the SWK beyond those who were originally considered “champion” providers

Having institutional support for CAM increased interest in the SWK beyond the obvious CAM experts. Requests for the SWK came not just from those with a professional focus on CAM, such as yoga instructors, acupuncturists and psychologists who specialize in meditative practices. Some providers wished to increase awareness of the SWK among like-minded colleagues, in the hopes that they too would begin to use it as a clinical tool. Provider networking proved effective for increasing SWK demand and building broad support and knowledge about the SWK at particular sites. On several occasions, colleagues of the original champion providers contacted the SWK team to request copies to disseminate within other clinics or settings. Requests for the SWK came from providers including occupational therapists, social workers, and primary care doctors with less obvious CAM specialization.

In other cases, providers distributed the SWK to their colleagues as a training resource for those who were less familiar with CAM. For example, the lead provider of an Opioid Safety Initiative distributed the SWK to providers at eight regional facilities as part of training to promote non-opioid pain management strategies. In two other instances, SWKs were disseminated to providers at conferences that promoted whole health and patient-centered care.

##### Provider autonomy allowed in dissemination influenced SWK dissemination and implementation, with variations in autonomy across sites

Allowing individual providers to develop their own plans for dissemination appeared to increase buy-in, with some providers even finding unanticipated uses for the SWK. While providers received “tip sheets” and support from the SWK team, individual providers chose which patients to offer the SWK, in what context, and what types of conversations and follow-ups they would attempt given the context. Various plans included dissemination in one-on-one sessions, group meetings, and larger educational programs such as health fairs.

Conversely, some providers felt their decision making autonomy was hindered by real or perceived institutional policy, and they were hesitant to disseminate the SWK. Some champion providers reported wanting more explicit approval from their institutions, despite the SWK being a VA-based project. For example, one provider delayed dissemination while she checked with her manager. Two others expressed reluctance to disseminate until checking with department heads, and then did not follow through. This delay between expression of interest and local approval may dissipate provider interest.

## Discussion

Many lessons from the SWK project support the existing literature about factors that support or hinder effective dissemination and implementation. Passive dissemination is common and lower cost. However, as previous evidence has shown [2], it does not lead to a wide-scale uptake. The threshold initiative for patients to take the SWK from the waiting room was low, but use of the tool and follow-up were also presumed low in this situation.

Reaching patients through their providers is a more effective means of implementing a large scale, sustainable education dissemination program. However, handing tools to providers with little commitment or knowledge of the subject matter may not fare much better than passive dissemination. Some providers had trouble finding time or the appropriate context to discuss the SWK within a clinical visit, and some had little previous knowledge of CAM, or connection to colleagues with this expertise.

The implementation phase addressed many known determinants of successful dissemination. In terms of system antecedents to innovation [5], the VA is large and mature enough to incorporate new tools and ideas. Many selected sites also had institutional CAM support. The SWK was most successful in settings with both champion providers and existing infrastructure to support CAM, which is likely related to the institutions’ leadership. By targeting champions, we sought out providers with preexisting CAM knowledge. This was effective because the provider community was engaged with the message, and the educational tool fit into the broader local context. Champion providers were more likely to communicate to Veterans that they were on board with CAM, and available for follow-up questions or for assistance integrating CAM into overall healthcare.

Other factors can improve dissemination after system antecedents are in place [5], including allowing providers to dictate the dissemination method. Our results show the value of this practice. Providers need to be engaged with education, organizational supports, and peer communication to increase awareness and value of educational tools. However, they also need to adapt dissemination to fit the confines of their particular duties and time commitments, addressing needs of the population they serve.

Results of this project also illustrated that good internal communication improves dissemination efforts [5]. Not all providers can begin as “champion” providers in a given area. SWK use spread through provider-initiated dissemination to colleagues, including to providers who did not specialize in CAM. Having the SWK distributed by known colleagues with broader training may increase provider interest in using the SWK first as a learning tool and then as a teaching tool for their patients. When introducing any new health education tool, optimal success may require culture change and infrastructure developments as well as opportunities for collaboration among healthcare providers.

While our experience may inform any educational healthcare tool, some features may be unique to CAM-related interventions and the VA setting. Primary care doctors have expressed interest in having more information on CAM [8], which continues to spread within medical contexts [9, 10]. In terms of the SWK project, VA CAM offerings have been expanding over the past decade [6], which likely has increased Veteran and VA provider interest. One published paper discussed lessons learned in disseminating CAM curricular initiatives within a medical education setting [11]. While the setting differs from ours, the authors’ experiences implementing CAM education support our recommendations and experiences. For example, they note that some faculty were resistant due to lack of time and not prioritizing CAM. However, they ultimately described successful CAM integration into the curriculum using many of the same tactics as the SWK project, including nurturing the organization’s leadership on the matter, increasing educational opportunities, and making CAM a part of their infrastructure by embedding it in other educational activities. Another study examined the integration of stand-alone CAM clinics into their broader communities and healthcare settings [12] and emphasized the importance of “visionary” champions within the centers as well as strong internal networking and communication.

### Limitations

Because the SWK was a clinical implementation project, we lacked empirical data from Veterans who received the SWK, especially during the implementation phase. Evidence of successful and unsuccessful dissemination strategies is based on the rate of survey return or on qualitative feedback from semi-structured interviews with providers. We obtained only limited data from Veterans about their actual use and adaptation of the tool. Evaluation of a dissemination and implementation strategy and its potential impact is best conducted by direct patient experience and observation. However, to maintain patient anonymity and allow for provider freedom in dissemination, and due to the national scale of the implementation project and the imperative to disseminate the SWK as quickly as possible, the information we present is based largely on provider experience and feedback.

## Conclusions and practice implications

Many of the lessons learned from the SWK project can be applied to the dissemination of any new educational tool within a healthcare setting. The results of the SWK project reiterate the importance of utilizing best practices for introducing educational tools within the healthcare context and the need for thoughtful, multi-faceted dissemination strategies. Systematic evaluation of future CAM education and implementation efforts should build on this report and expand our knowledge of best practices.

Of note, based on feedback received, the STAR Well-Kit is now available entirely online at www.warrelatedillness.va.gov/education/STAR.

## Abbreviations

CAM: Complementary and alternative medicine
OPCC & CT: Office of patient-centered care and cultural transformation
SWK: Star well-kit
VA-NJHCS: VA New Jersey Healthcare System
WRIISC: War related illness and injury study center
